# The long and the short of it: Adaptation of carbon uptake and metabolic flux to different daylengths

**DOI:** 10.1093/plphys/kiad523

**Published:** 2023-10-09

**Authors:** Alexandra J Burgess, Gustaf E Degen

**Affiliations:** Assistant Features Editor, Plant Physiology, American Society of Plant Biologists; Agriculture and Environmental Sciences, School of Biosciences, University of Nottingham Sutton Bonington Campus, Loughborough LE12 5RD, UK; Assistant Features Editor, Plant Physiology, American Society of Plant Biologists; Plants, Photosynthesis and Soil, School of Biosciences, University of Sheffield, Firth Court, Western Bank, Sheffield S10 2TN, UK

Plants grow in diverse environments: on freezing islands close to the Antarctic, in sweltering deserts, or in humid tropical rainforests. In contrast to animals such as migratory birds, they are sessile and must adapt to environmental changes, including differences in temperature, rainfall, or changes in daylength (photoperiod) throughout the seasons of the year. This ability to adapt can be controlled by diverse mechanisms and could provide potential targets to improve crop performance.

The photoperiod is determined by seasonal or latitudinal variation, with increasing distance from the equator determining the magnitude of variation in day length experienced. This variation impacts plant growth by altering photosynthetic physiology and metabolic fluxes. However, relatively speaking, plants grow faster under shorter photoperiods, indicating that short day (SD) plants have adapted mechanisms to compensate for fewer daylight hours. The metabolic pathways in which these adaptations occur are still unknown but are expected to encompass traits associated with an increase in light-use efficiency and allocation of fixed carbon toward growth.

Metabolic fluxes are measured using metabolic flux analysis (MFA), where reaction rates are determined using stable and radioisotopes. However, accurately measuring reactions is confounded by the varying rates at which they occur. For example, flux through Calvin-Benson-Bassham cycle intermediates occurs within subseconds, sugars within hours, and storage pools over the timescale of days. Therefore, traditional MFA is unsuitable for analysis over such varying timeframes or when there is a high degree of compartmentation (Allen et al. 2009). Fortunately, recent advances in ^13^CO_2_ time-course labeling and computational modeling have led to the development of isotopically nonstationary MFA (INST-MFA) to study in vivo carbon fluxes (Cheah and Young 2018; Chu et al. 2022). INST-MFA describes the time-dependent labeling of network metabolites, concurrently adjusting flux and pool size parameters and therefore providing a more flexible and sensitive approach to estimating fluxes in autotrophic systems. One advantage of INST-MFA is that it can identify features of metabolic reactions that cannot be obtained through other approaches, including the identification of subcellular compartmentation of reactions and relative differences in pool sizes.

In this issue of *Plant Physiology*, Xu et al. (2023) determine the response of *Camelina sativa* to differences in daylength using growth and gas exchange measurements combined with INST-MFA (Fig. 1). Camelina has a wide geographic distribution, thus making it a useful model to study how plants adapt to changes in the photoperiod. Xu et al. (2023) found that adaptation occurs through a combination of different mechanisms. In terms of gas exchange, SD plants exhibited a higher photosynthetic rate (21% greater than long day [LD] plants) and a lower respiration in the light. This was attributed to an increased CO_2_ concentration around Rubisco combined with a reduced flux through the glucose 6-phosphate shunt. SD plants exhibited higher shoot:root ratios and thinner leaf laminas to maximize overall photosynthetic area. Finally, the rate of starch synthesis was enhanced in SD plants, representing a larger carbon storage pool for maintenance during the longer night. A higher rate of starch synthesis during SD conditions is a consistent finding across a wide range of C3 species (e.g. Zeeman et al. 2004; Sulpice et al. 2014) but contrasts with sucrose synthesis in which there is not a consistent response across C3 plants according to daylength (e.g. Logendra et al. 1990; Mugford et al. 2014).

**Figure 1.**
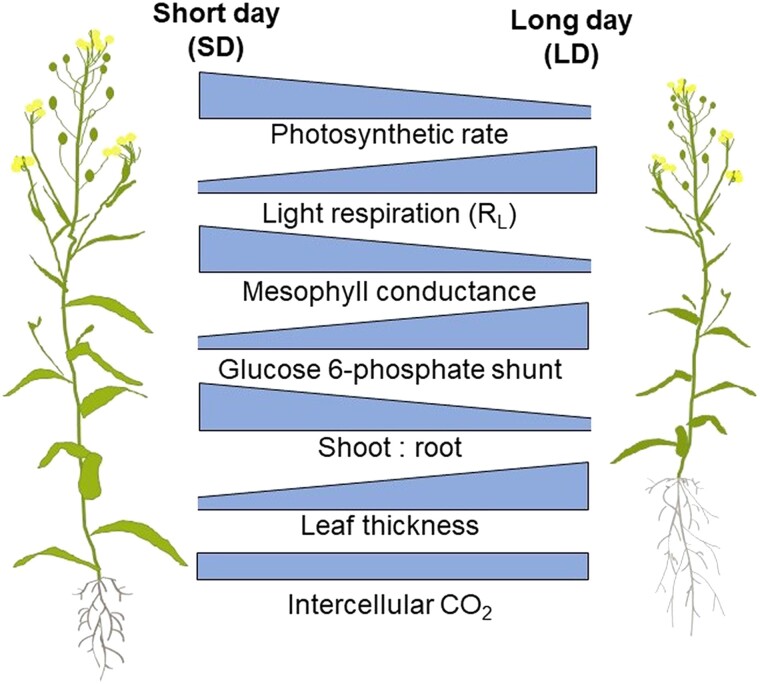
Overview of the impact of daylength on carbon metabolism and physiology of *Camelina sativa*.

To complete the picture of how Camelina plants adapt to short days, the authors used INST-MFA to compare SD with LD plants. A higher discrepancy for ^13^CO_2_ was witnessed in SD plants, indicating a reduced diffusion resistance to CO_2_ uptake. Similarly, a more negative δ^13^C confirmed a higher mesophyll conductance within SD plants. Together, a reduced diffusion resistance combined with a higher mesophyll conductance contributed to the higher photosynthetic rate in SD plants and led to the maintenance of similar intercellular CO_2_ values regardless of day length. For Camelina grown under SD conditions, the larger total amino acid levels were partitioned within a smaller active pool and a larger storage (i.e. vacuolar) pool, relative to LD plants. However, measured changes in pool sizes of some metabolites, including glycine, were inversely correlated with the ^13^C and ^2^H labeling patterns, indicating metabolically inactive pools located across multiple compartments.

In conclusion, the study performed by Xu et al. (2023) provides an insight into the adaptive strategies that C3 plants may use to counteract the effect of a change in day length. Results indicate how the adjustments and compartmentation of metabolite pools lead to an enhancement of photosynthetic productivity under SDs. These mechanisms provide ecological adaptation to different growth locations and seasons but also represent potential targets for improving carbon metabolism as part of yield improvement programs.

## Data Availability

There are no new data associated with this article.
